# Accuracy of blind approach and ultrasound–guided techniques for pudendal nerve block in cadaveric cats - a pilot study

**DOI:** 10.1007/s11259-026-11070-1

**Published:** 2026-02-05

**Authors:** Mariana Melânia Cristofolini, Henrique Zem Chequin Sprengel, Julia Milczewski Vilani, Viviane Luize Bosak, Marcello Machado, Ricardo Guilherme D’Otaviano de Castro Vilani

**Affiliations:** 1 Department of Veterinary Medicine, Federal University of Paraná, Rua dos Funcionários, 1540, Juvevê, Curitiba, Paraná Brazil; 2 Anatomy Department, Biological Science Sector, Federal University of Paraná, Jardim das Américas, Curitiba, PR Brazil

**Keywords:** Cadaver study, Feline, Regional anesthesia, Urethral obstruction

## Abstract

This blinded, randomized and prospective cadaveric pilot study compared the accuracy of a blind approach for pudendal nerve block (PNB–B) with that of the ultrasound–guided technique (PNB–US) in cats. Two male cat cadavers were used for a preliminary anatomical study to identify palpable bony landmarks, while eight additional cadavers were employed for the experimental phase. Each hemipelvis was randomly assigned to one of the two techniques: PNB–US, performed according to the deep dorsolateral method described by Adami et al. (J Feline Med Surg 15:901–907, 2013), or the PNB–B, developed based on the preliminary anatomical findings, identifying the application point laterally to the sacrococcygeal joint. Block accuracy was assessed by dye spread, considering the nerve branches stained in each approach. The success rate was 87.5% (7/8) for PNB–B and 75% (6/8) for PNB–US. In the injections considered accurate in PNB–B, dye was deposited at the point of confluence of sacral nerve branches forming the pudendal nerve, while in PNB–US the pudendal nerve was stained near the exit of the pelvis. The ischial nerve was inadvertently stained in 75% (6/8) of PNB–B and 25% (2/8) of PNB–US injections. These findings indicate that PNB–B can be considered an effective alternative to the ultrasound–guided technique, although, in clinical settings, it may produce motor blockade due to unintended ischial nerve involvement. The results provide preliminary data to support future in vivo clinical studies aimed at confirming the safety and analgesic efficacy of this novel blind technique in cats.

## Introduction

Locoregional anesthesia provides analgesia and reduces general anesthetics and opioids requirements (Mosing et al. 2010; Congdon et al. 2017; Pratt et al. 2020). It is recommended for all patients, particularly those in whom minimizing anesthetic risk is essential, such as individuals with metabolic or hemodynamic compromise. The coccygeal epidural block promotes urethral relaxation and analgesia for feline urogenital procedures (O’Hearn and Wright 2011; Pratt et al. 2020) but has potential complications and contraindications (Ferreira 2018; Dancker et al. 2019) and often shows limited success rates, frequently requiring multiple attempts (Pratt et al. 2020).

The pudendal nerve block (PNB) is a more selective alternative for analgesia and relaxation of the penis and urethra, as the pudendal nerve supplies the musculature and skin of the anus, perineum, urethra, and penis (Uemura 2015; König et al. 2016). Ultrasound–guided PNB (PNB–US) has shown clinical efficacy in cats (Adami et al. 2014), and an alternative approach has also been described (Briley et al. 2022). However, no studies have compared PNB-US with a blind approach (PNB-B), which may be useful in clinical practice.

Given limited ultrasound equipment availability and trained professionals, evaluating PNB-B feasibility is necessary. Therefore, the aim of this pilot study is to compare the feasibility and accuracy of PNB-B with PNB–US in cats through a prospective cadaveric study, providing preliminary data for future clinical research.

## Materials and methods

### Animals

Ten male mixed–breed cat cadavers, either frozen (thawed within 72 h), refrigerated (removed from the refrigerator 12 h before use) or fresh (within six hours of death) were enrolled in the study. Cadavers were obtained from the veterinary teaching hospital of the Federal University of Paraná (Curitiba, PR, Brazil), where the study was conducted. All animals died of unrelated causes and were donated, through owner written consent, for use in teaching and research. According to institutional policy, ethical approval was not required for cadaveric studies. The study was reported following the AQUA guidelines (http://www.eba.cm.uj.edu.pl/aqua).

**Fig. 1:**
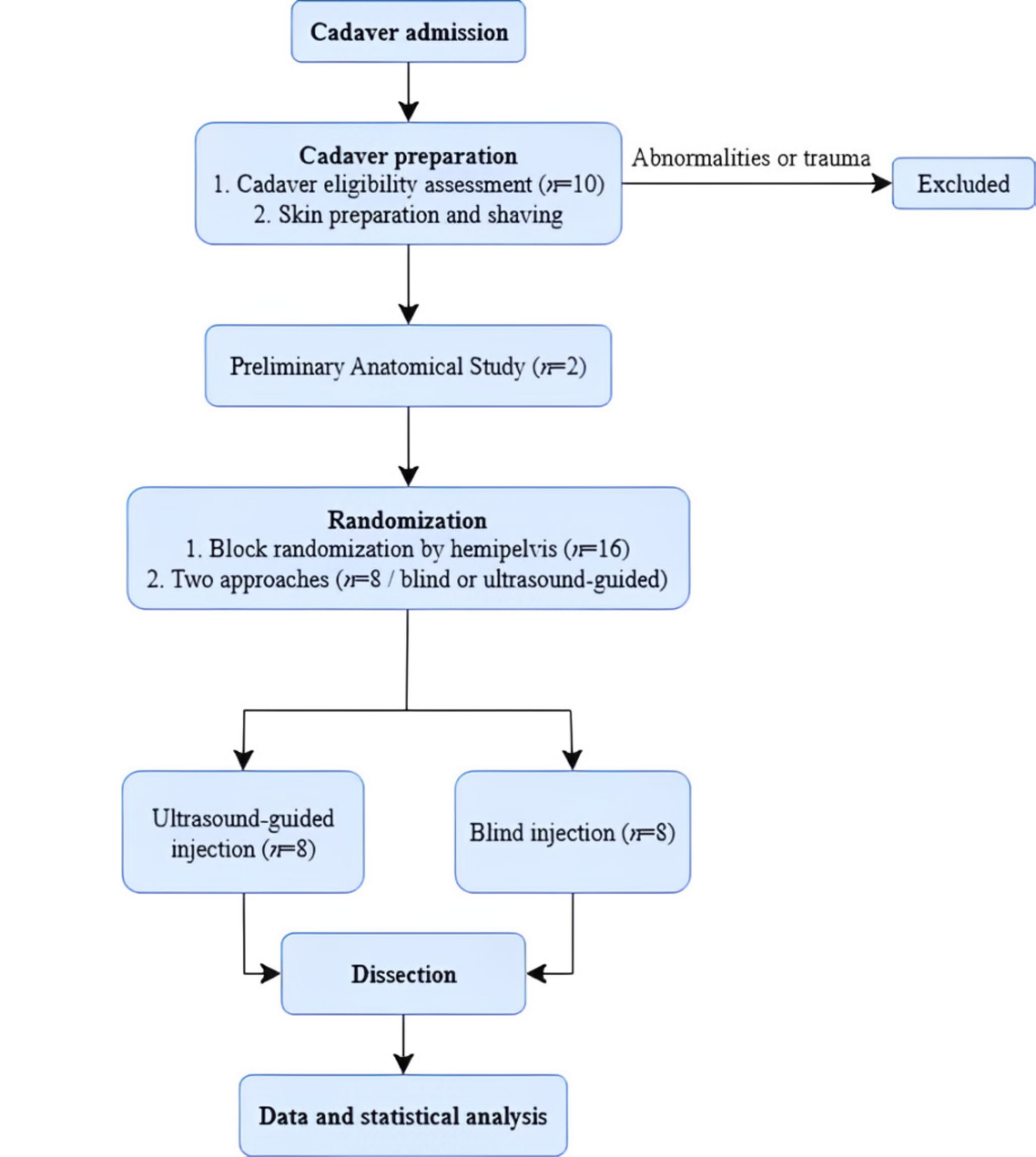
Flowchart illustrating the study protocol, including cadaver preparation, preliminary anatomical study, randomization by approach, ultrasound–guided and blind injections, exclusion criteria and dissection before statistical analysis

Two cadavers were used for a preliminary anatomical study. The remaining eight were used in the experimental trial. Animals showing signs of trauma or obvious anatomical abnormalities in the pelvic region were excluded (Fig. 1).

### Preliminary anatomical study

The aim was to determine the PNB–B insertion point and to recognize potential risks of iatrogenic injury. A feline skeleton was used to identify palpable bony landmarks that would guide needle placement. One formalin–fixed cadaver preserved was used to identify the pudendal nerve and adjacent structures. A second fresh cadaver was used for ultrasound study and for preliminary testing the developed blind approach. An experienced veterinary anatomist (MM) performed both dissections.

### PNB techniques

Each hemipelvis was randomly assigned by block randomization (http://www.random.org) to two equal–sized groups: ultrasound–guided PNB (US group; *n* = 8) or blind PNB (B group; *n* = 8). Cadavers were shaved in the lumbar and sacral areas and positioned in ventral recumbency with hind limbs extended caudally in a frog–leg position. Blocks were performed by the same veterinary anesthesiologist (MMC) and always started on the right hemipelvis to limit bias related to hand dominance.

For PNB–US, a deep dorsolateral approach was used (Adami et al. 2013) with a 6–12 MHz linear probe (LOGIQ F6 R2; GE Medical Systems CO. LTD., China). A 20–gauge Tuohy needle (BD Perisafe; Becton Dickinson Indústrias Cirúrgicas Ltda, PR, Brazil) was inserted craniocaudally to the dorsal aspect of the pelvic urethra, where 0.1 mL kg^− 1^ of 1% methylene blue dye (Drogavet, PR, Brazil) was injected per site.

**Fig. 2:**
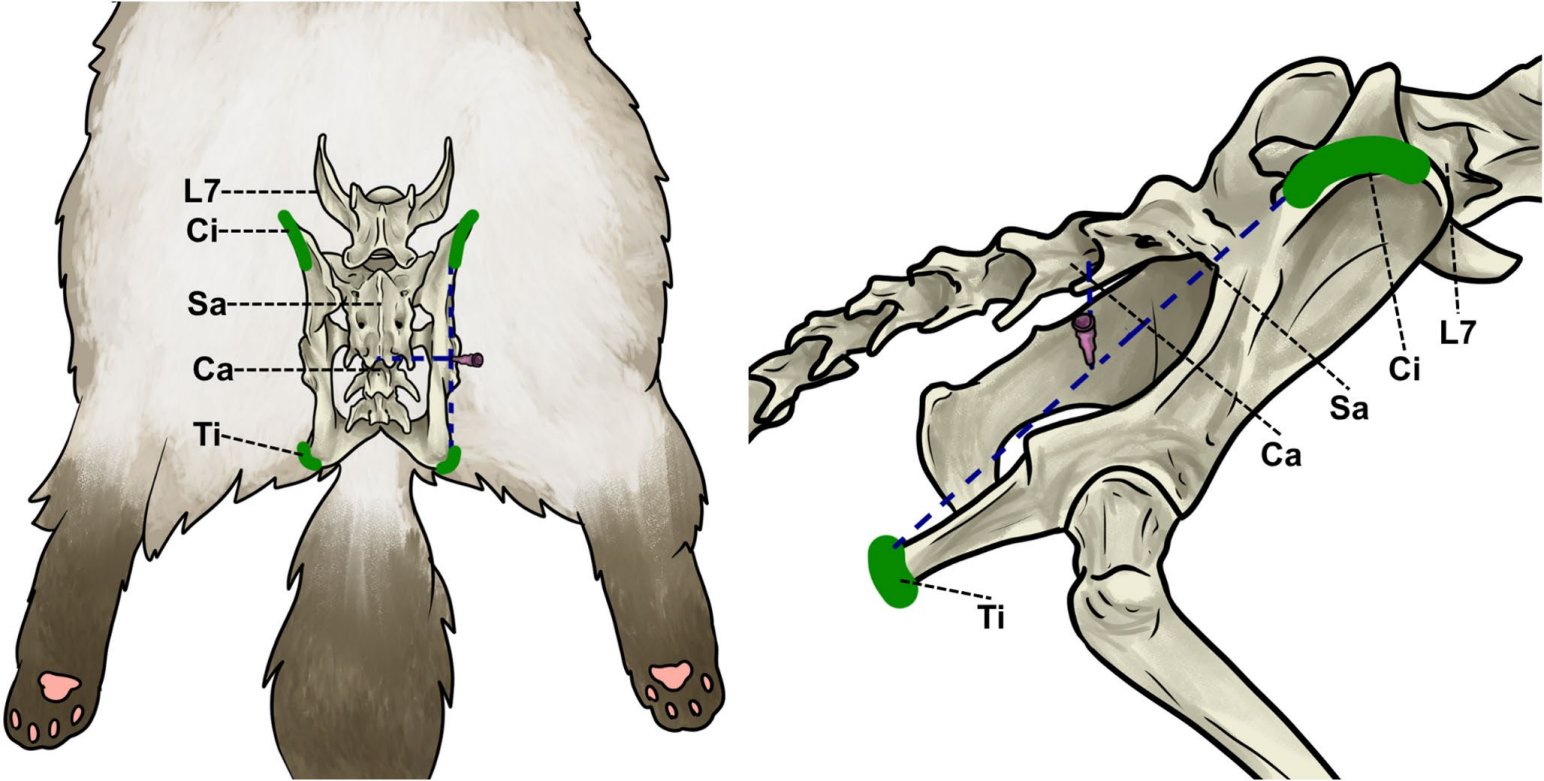
Schematic representation of blind approach for pudendal nerve block in a male cat. Ci, iliac crest; Ti, ischial tuberosity; L7, seventh lumbar vertebra; Sa, sacrum; Ca, first caudal vertebra. Needle angulation and insertion site is represented by the purple needle

Palpable bony landmarks identified in the preliminary study, mainly the sacrum, ischial tuberosity, and iliac crest, were used to guide PNB–B. With the thumb and middle fingers on the iliac crests, the index finger traced the midline to identify the last lumbar spinous process, the depression between the last lumbar vertebra and sacrum, and the spinous processes of the sacral vertebrae and first caudal vertebra. The joint between the sacrum and first caudal vertebra was defined as the Sa–Ca point. An imaginary line between the ischial tuberosity and iliac crest (Ti–Ci line) was drawn, and a perpendicular line from the Sa–Ca point intersected the Ti–Ci line (Point A), approximately 2.5 cm from the midline. A 20 mm × 0.55 mm hypodermic needle (Labor Import, SP, Brazil) was inserted at Point A, directed medioventrally at a 45° angle, where 0.1 mL kg^− 1^ of 1% methylene blue was deposited (Fig. 2).

### Dissection

Pelvic dissection began within 15 min after block completion, always starting on the right hemipelvis. An experienced anatomist (MM), blinded to the technique, performed all dissections. Skin incisions were made around the limb root, and the hind limb was removed by transecting musculature and opening the hip joint. Partial hemipelvectomy exposed the pelvic plexus, pudendal nerve, and adjacent structures. The procedure was mirrored on the left side.

### Data analysis

Block success was assessed using a visual scale ranging from 0 to 5, based on the intensity of pudendal nerve staining, where 0 indicated absence of dye and 5 indicated maximal dye impregnation, as determined by an experienced veterinary anatomist blinded to the technique (MM). Blocks were considered ineffective when scores were ≤ 1. Dye dispersion was measured along the craniocaudal (C–C) and dorsoventral (D–V) projections, and the stained nerve branches were evaluated. Recorded variables included body weight, spine length, body condition score according to Freeman et al. (2011) 1–9 scale, block execution time, and dissection time. Potential iatrogenic complications involving the rectum, urethra, or vasculature were also documented.

Data were analyzed using commercial software (SigmaPlot for Windows, version 12.0; Systat Software Inc., Germany). Normality was assessed with the Shapiro–Wilk test. Given the pilot nature of the study and limited sample size, no inferential statistical testing was performed. Results are presented as mean ± standard deviation for parametric variables and as median (range) for non–parametric ones.

For parametric continuous variables, group differences are expressed with 95% confidence intervals (CI) of the mean difference. For binary outcomes, differences in proportions between groups were reported with 95% CI, estimated using the standard error of the difference in proportions. Confidence intervals were not calculated for non–parametric variables. All results are descriptive and should not be interpreted as confirmatory statistical evidence.

## Results

### Anatomical study

The preliminary anatomical study identified the PNB-B target at the post-foraminal convergence of the pudendal nerve, formed by ventral sacral branches running caudoventrally from the pelvis. The pelvic plexus was identified lateral to the middle rectum, dorsal to the prostate, and within pelvic fascia with blood vessels. The pelvic nerve was observed between the sacral and pelvic plexuses, cranial to the pudendal nerve. The pelvic urethra, prostate, and bulbourethral glands were also identified.

### Experimental study

Sixteen nerve blocks were evaluated (B, *n* = 8; US, *n* = 8). No cadavers were excluded after randomization. Body conformation was similar between groups: mean body weight was 3.60 ± 0.58 kg in B and 3.70 ± 0.53 kg in US (95% CI − 0.64 to 0.44 kg); spinal lengths was 54 (51–58.5) cm in B and 52.5 (51–58.9) cm in US; and body condition scores were 4.25 ± 1.58 in B and 5.00 ± 0.76 in US (95% CI − 2.11 to 0.61).

The time required to perform the blocks was 43.5 (30–60) seconds in the B group and 318.5 (165–938) seconds in the US group. The C–C dye spread was 3.13 ± 0.60 cm in B and 2.72 ± 0.58 cm in US (95% CI − 0.17 to 0.99 cm), while the D–V spread was 1.30 ± 0.51 cm and 1.69 ± 0.93 cm (95% CI − 1.12 to 0.34 cm), respectively.

The success rate of the nerve block was 7/8 injections (87.5%) in B and 6/8 (75%) in US (95% CI − 0.25 to 0.50%). In successful B group injections, dye was deposited at the convergence of the sacral nerve branches forming the pudendal nerve shortly after their sacral emergence. In the US group, the pudendal nerve was stained more caudally, near the pelvic outlet, without involvement of other lumbosacral plexus branches. The ischial nerve was stained in 75% (6/8) of B group injections and in 25% (2/8) of US group injections (95% CI 0.08 to 0.92%). No iatrogenic complications were observed in any procedure.

## Discussion

The peripheral nerves supplying the pelvic region and hind limbs originate from the lumbosacral plexus, formed by the ventral branches of the last lumbar and sacral spinal nerves (L4–S3) (Thomson and Hahn 2012; Evans and Lahunta 2013; Uemura 2015; König et al. 2016). The pudendal nerve is a mixed nerve derived from the ventral branches of the three sacral spinal nerves (Evans and Lahunta 2013). In cats, it typically receives fibers from S2 and S3 (Nickel et al. 2004; Schaller 2007), with occasional contribution from S1 (Ghoshal 1986). Although pudendal formation patterns could not be assessed due to difficulty in identifying communicating branches once stained and limited dissection time before dye dispersion, this limitation did not affect injection site identification, as the S1, S2, and S3 nerves lie in close proximity at their pelvic emergence.

The preliminary anatomical study using skeleton and cadaver enabled identification of a PNB–B without apparent iatrogenic injury. Palpation of anatomical landmarks allowed the construction of reference lines to guide needle insertion. The needle length was selected based on the estimated thickness of the skin and epaxial musculature required to reach the target site.

Anatomically, the pudendal nerve follows an oblique course within the pelvic cavity, running caudoventrally toward the pelvic outlet, where it divides into the caudal rectal, perineal, and dorsal penile nerves (Ghoshal 1986; Evans and Lahunta 2013; Singh 2018). In this study, PNB-B targeted the pudendal nerve cranial to these divisions, ensuring blockade of sensory branches supplying the penis (Evans and Lahunta 2013). Although effective in 87.5% of cases, PNB–B was not selective. Unintended staining of neighboring lumbosacral plexus branches, including the ischial nerve, occurred in 75% of injections. While not considered a serious complication, motor block should be anticipated as a likely outcome when using the technique. Using lower concentrations of local anesthetics may help minimize the duration of motor impairment without compromising sensory block (Martin-Flores 2013).

Clinically, PNB–US has been reported to provide adequate perioperative analgesia in cats undergoing perineal urethrostomy (Adami et al. 2014; Briley et al. 2022), but requires substantial ultrasonographic knowledge and operator expertise. The success rate observed in this study (75%) may reflect this technical complexity and is consistent with previous reports in cats and dogs (Adami et al. 2013; Briley et al. 2022; Zumstein et al. 2025). Achieving an adequate sonographic window for pudendal nerve visualization is challenging and increases execution time compared to PNB-B. In the study by Adami et al. (2013), prior urethral catheterization may have facilitated urethral visualization, which commonly is challenging. However, in clinical practice, such catheterization is often impractical prior to nerve block, especially when the block is intended to allow the procedure itself. Similarly, performing the block in the presence of an imaging specialist may have contributed to the success of the technique. While more challenging and time-consuming than PNB-B, PNB-US allows monitoring of needle trajectory and anesthetic spread, potentially increasing selectivity and potentially reducing the risk of complications associated with non–guided techniques (Zumstein et al. 2025).

This pilot study revealed that PNB–B was effective in 7 out of 8 injections and could be performed rapidly, without evidence of iatrogenic complications, indicating promising applicability. The use of easily palpable bony landmarks likely contributed to the speed and accuracy of the technique, which is simple and low–cost. However, its clinical safety and effectiveness must still be validated in vivo.

This study has limitations. Frozen-thawed cadavers may not fully replicate biomechanical and acoustic properties of live tissues. The fact that the veterinary anesthesiologist performing the procedures had only two years experience may introduce bias in the execution of PNB–US, potentially affecting outcomes such as success rate and injection time. The visual scale used to define block success is subjective and may not necessarily reflect adequate sensory blockade; therefore, clinical studies are required to confirm the effectiveness of the block. Finally, the unintended staining of the ischial nerve observed in PNB–B should be considered a potential iatrogenic effect, warranting caution in clinical application.

Our study suggests that the PNB-B may be considered effective, although in clinical situations it may induce motor block due to unintended ischial nerve involvement. These preliminary findings provide pilot data to guide future in vivo clinical studies, which are necessary to validate the applicability of the technique and to assess its safety and analgesic efficacy.

## Data Availability

The datasets generated during and/or analysed during the current study are available from the corresponding author on reasonable request.
